# Protective Effects of Wheat Peptides against Ethanol-Induced Gastric Mucosal Lesions in Rats: Vasodilation and Anti-Inflammation

**DOI:** 10.3390/nu12082355

**Published:** 2020-08-07

**Authors:** Lanlan Yu, Ruijun Li, Wei Liu, Yalin Zhou, Yong Li, Yong Qin, Yuhan Chen, Yajun Xu

**Affiliations:** 1 Department of Nutrition and Food Hygiene, School of Public Health, Peking University, Beijing 100083, China; putaoeternal@163.com (L.Y.); aiesecarrow@163.com (R.L.); liuwei19560912@163.com (W.L.); zhouyalin2017@163.com (Y.Z.); 15895977095@163.com (Y.L.); qinyong0520@163.com (Y.Q.); cyhan_ss@163.com (Y.C.); 2 Beijing Key Laboratory of Toxicological Research and Risk Assessment for Food Safety, Peking University, Beijing 100083, China

**Keywords:** wheat peptides, gastroprotection, gastric mucosal blood flow, nuclear factor kappa B, inflammation

## Abstract

Alcohol consumption increases the risk of gastritis and gastric ulcer. Nutritional alternatives are considered for relieving the progression of gastric mucosal lesions instead of conventional drugs that produce side effects. This study was designed to evaluate the gastroprotective effects and investigate the defensive mechanisms of wheat peptides against ethanol-induced acute gastric mucosal injury in rats. Sixty male Sprague–Dawley rats were divided into six groups and orally treated with wheat peptides (0.1, 0.2, 0.4 g/kgbw) and omeprazole (20 mg/kgbw) for 4 weeks, following absolute ethanol administration for 1 h. Pretreatment with wheat peptides obviously enhanced the vasodilation of gastric mucosal blood vessels via improving the gastric mucosal blood flow and elevating the defensive factors nitric oxide (NO) and prostaglandin E2 (PGE2), and lowering the level of vasoconstrictor factor endothelin (ET)-1. Wheat peptides exhibited anti-inflammatory reaction through decreasing inducible nitric oxide synthase (iNOS) and pro-inflammatory cytokines tumor necrosis factor α (TNF-α), interleukin (IL)-1β, IL-6, and increasing trefoil factor 1 (TFF1) levels. Moreover, wheat peptides significantly down-regulated the expression of phosphorylated nuclear factor kappa-B (p-NF-κB) p65 proteins in the NF-κB signaling pathway. Altogether, wheat peptides protect gastric mucosa from ethanol-induced lesions in rats via improving the gastric microcirculation and inhibiting inflammation mediated by the NF-κB signaling transduction pathway.

## 1. Introduction

The destruction of gastric mucosal integrity is a result of an imbalance between defensive factors (mucus, bicarbonate, nitric oxide (NO), prostaglandins, and gastric mucosal blood flow) and the aggressive factors (hydrochloric acid and pepsin) [1]. Injurious agents such as *Helicobacter pylori*, non-steroid anti-inflammatory drugs (NSAIDs), alcohol abuse, and stress are the main factors responsible for gastric mucosal lesions creating fall in gastric mucosal blood flow and an impairment of mucosal defensive mechanisms [2,3]. Excessive alcohol consumption results in necrotic lesions in the gastric mucosa inducing acute hemorrhagic gastritis and ulcerative damage [4,5]. If untreated, gastritis progresses to gastric ulcer and carcinoma [6]. 

The pathophysiologic mechanisms of ethanol-induced gastritis have not been completely established [7]. However, cumulative evidence suggests that a significant destruction of the gastric mucosal microcirculation and epithelial cell proliferation results in ischemia, hypoxia, and reactive oxygen species (ROS) generation, and thus contributes to the inflammatory responses via the nuclear factor kappa B (NF-κB) cascade [8]. Ethanol intake could destroy the integrity of gastric mucosal surface through lowering gastric mucosal blood flow, which is associated with reductions in major endogenous defensive factors for NO and prostaglandin E2 (PGE2) [9,10,11]. In addition, activated NF-κB stimulates multiple downstream effectors of inflammatory mediators such as enzymes for inducible nitric oxide synthase (iNOS) and cytokines such as tumor necrosis factor α (TNF-α), interleukin (IL)-1β and IL-6, which in turn may magnify gastric damage after ethanol exposure [12,13].

Traditional clinical methods, such as proton pump inhibitors, antacids, and H2 receptor antagonists, cause many adverse reactions [14]. Bioactive peptides are defined as peptide sequences in proteins, with the characteristics of high selectivity, safety, and effectiveness, when used as non-drug functional foods. Myriad activities in peptides exert beneficial effects on bodily function and have health benefits in controlling disorders such as hypertension, diabetes, obesity, and cancer [15,16].

Wheat peptides, derived from wheat protein hydrolyzation by proteolytic enzymes, possess new nutritional, functional, or biological properties than wheat protein, including proven anti-oxidant and anti-inflammatory effects [17,18]. Furthermore, wheat peptides have been shown to promote vasodilation activity through increasing the secretion of NO in human umbilical vein endothelial cells [19]. Previous studies demonstrated that wheat peptides administration could protect against non-steroidal anti-inflammatory drug (NSAID)-induced gastrointestinal damage by suppressing the expression of pro-inflammatory cytokine in rats [20]. In addition, management with wheat peptides could restrain ethanol-induced gastric mucosal damage through the mechanisms of anti-oxidation, anti-inflammation, and pro-survival in human gastric epithelial cell line (GES-1) [21]. However, the effects of wheat peptides on gastric blood flow and the specific anti-inflammatory mechanism have not been fully elucidated in a rat model of acute ethanol-induced gastric mucosal lesions.

Hence, the purpose of this study was to evaluate the potential gastroprotective role of wheat peptides against acute ethanol-induced gastric lesions. Moreover, this is the first study to investigate the effects of wheat peptides on gastric mucosal blood flow and inflammatory responses modulated by the NF-κB signaling pathway in an ethanol-induced gastric injury model of rats. 

## 2. Materials and Methods 

### 2.1. Meterials

Wheat peptides were obtained by gluten hydrolyzation. The manufacturing process of wheat peptides was as follows: the gluten powder was mixed with a certain amount of 53 °C hot water by the ratio of liquid to material as 100:1 (kg/L), stirred uniformly. Then, flake caustic was added to the mixture solution to adjust the pH to approximately 9.0–9.3 alongside an appropriate amount of enzyme to react (neutral protease: alkaline protease = 1:2, holding 50 ± 2 °C). After reaching the predetermined time, the waste residue and destroyed enzyme (inlet temperature 121 ± 5 °C; outlet temperature 60–65 °C) were removed from the resulting solution of wheat oligopeptide. Lastly, the concentrated solution received ultra-high temperature instant sterilization to kill the microorganisms, after which it was treated with spray drying.

### 2.2. Animals and Treatment

Sixty healthy male Sprague–Dawley (SD) rats weighing 180–200 g were provided by the Department of Laboratory Animal Science of Peking University (Beijing, China, SCXK 2016-0010). The animals were housed under controlled laboratory conditions (temperature (23 ± 2 °C), humidity (55 ± 5%), and a 12/12 h light/dark cycle), and had free access to distilled water and a standard diet for 7 days acclimatization before starting the experiments. All of the animal experiments were approved by the Ethic Committee of Peking University Health Science Center (approval code LA2017092) in accordance with the Regulations for the Administration of Affairs Concerning Experimental Animals (Beijing, China).

Rats were randomly divided into 6 groups with 10 animals in each group as follows: (1) normal control group (NC); (2) ethanol model control group (EC); (3) positive control (OME, omeprazole); (4) low dose of wheat peptide (LWP); (5) medium dose of wheat peptide (MWP); (6) high dose of wheat peptide (HWP). The rats were orally dosed with the vehicle (distilled water, 10 mL/kg), omeprazole (20 mg/kgbw), or wheat peptides at three doses (0.1, 0.2, or 0.4 g/kgbw). 

### 2.3. Examination of GMBF and Ethanol-Induced Gastric Mucosal Damage

After 4 weeks intervention, all the animals were fasted for 24 h with free access to water. Animals in the wheat peptides intervention group were orally administrated with 1 mL of absolute ethanol, with animals in NC group given 1 mL of distilled water.

After 50 min of ethanol administration, gastric mucosal blood flow (GMBF) was measured in each animal. After ether anesthesia, the stomach was exposed by a cut along the midline of the abdomen. Laser doppler blood flowmeter and miniature surface probes (moorVMS-LDF; Moor, Devon, UK) were used to measure the gastric mucosal blood flow after a 0.3 cm opening was made in the anterior wall of the stomach. The laser probe was placed on four points of the stomach, including the gastric fundus, the gastric antrum, the midpoint of the lesser gastric curvature, and the midpoint of the greater gastric curvature. The blood flow of every point was recorded for three times, each measuring 15 s, in each rat. The relative signals were transformed into blood perfusion units (BPUs) and recorded with a computer. The stable signals were selected to analyze and calculate the mean value of the four points of every gastric sample with matching software moorVMS-PC v3.1 (Moor, Devon, UK). 

After 1 h of ethanol administration, blood was collected from the femoral artery, and the serum was centrifugated at 3000 rpm/min for 10 min and stored at −80 °C until further analysis, after which the animals were sacrificed by cervical dislocation. The stomachs were removed for macroscopic and histopathologic analysis. 

### 2.4. Macroscopic Assessment

For evaluating the extent of gastric mucosal lesions, the removed stomachs were opened along the greater curvature and completely rinsed with cold saline for 30 min. After that, the drained and flattened stomach samples were photographed. Then, a vernier caliper was used to measure the length and width of lesions in the flattened stomach samples. Gastric mucosal damage was expressed as an injury index (Table 1).

### 2.5. Histopathological Analysis

After the general examination, gastric tissue was fixed in 10% formalin solution for 24 h. Tissue samples were dehydrated by alcohol, embedded in paraffin, and sectioned (5 μm thickness). The paraffin blocks were stained with hematoxylin and eosin (H&E) and examined under the fluorescence microscope (E400; Nikon, Tokyo, Japan) to evaluate gastric morphology.

### 2.6. Determination of Inflammation Cytokines Levels

The gastric tissue samples were weighed, shredded, and then homogenized in an ice-cold phosphate buffer saline (PBS). The proportion of gastric tissues and mixed PBS was 1:9. A part of homogenization buffer was centrifuged at 13,000× *g* at 4 °C for 10 min. The supernatants were collected for the determination of cytokines level and gastric tissue protein content.

In stomach homogenate, enzyme-linked immunosorbent assay (ELISA) kits were used to determine the secretion of tumor necrosis factor α (TNF-α) and interleukin (IL)-1β and IL-6 (ZSBABIO, Beijing, China), and Trefoil factor 1 (TFF1) (USCN, Wuhan, China) according to the manufacturer’s instructions, respectively. The results of TNF-α, IL-6, IL-1, and TFF1 were expressed as pg/mg tissue.

### 2.7. Determination of NO, ET-1 and PGE2 Levels

The level of nitric oxide (NO) in plasma was assessed as total nitrate/nitrite by a commercial assay kit based on Griess reaction (Nanjing JianCheng Bioengineering Institute, Nanjing, China). ELISA kits were used to measure prostaglandin E2 (PGE2) (MultiSciences, Hangzhou, China) in the stomach homogenate and endothelin (ET)-1 (ZSBABIO, Beijing, China) in plasma. All operations were carried out according to the manufacturer’s instructions strictly. The final results were expressed as umol/g of NO, pg/mL of ET-1 and pg/mg tissue (PGE2).

### 2.8. Determination of iNOS Levels

Gastric mucosal Nitric Oxide Synthase (NOS) activity was examined by commercial assay kits (Nanjing JianCheng Bioengineering Institute, Nanjing, China) based on a colorimetric method according to the guidelines of the manufacturer of the kits. Briefly, NOS catalyzes the production of NO from L-arginine with the assistance of molecular oxygen and calcium ions. During the reaction, generated NO was combined with nucleophilic substances and produced colored compounds. Then, the activity of iNOS was calculated based on the visual density measured at 530 nm via a Thermo Multiskan MK3 Automated Microplate Reader (Thermo-Labsystems, Franklin, MA, USA). The results of iNOS activity were expressed as U/mg protein. 

### 2.9. Western Blot for NF-κB p65 and p-NF-κB p65

Segments of gastric tissue were mixed with protein lysis buffer to extract the total protein of gastric cytoplasm and nuclear. The total protein concentration was quantified by a bicinchoninic acid method. First, 20 μg of the protein extraction were separated by 10% sodium dodecyl sulfate-polyacrylamide gel electrophoresis (SDS-PAGE) and subsequently transferred to polyvinylidene fluoride membranes (PVDF; Millipore, Billerica, MA, USA). Each membrane was blocked with 5% non-fat milk in Tris-buffered saline containing 20% Tween 20 for 4 h at room temperature. Then, the membrane was incubated with primary antibodies against nuclear factor kappa-B (NF-κB) p65 (#8242; CST, Danvers, MA, USA), phosphorylated nuclear factor kappa-B (p-NF-κB) p65 (#3033; CST, Danvers, MA, USA), and β-actin (ab8227; Abcam, Cambridge, UK) at 4 °C overnight. Horseradish peroxidase-linked goat anti-rabbit Immunoglobulin G (IgG) secondary antibodies (ab6721; Abcam, Cambridge, UK) were used to incubate the membranes for 4 h. The horseradish peroxidase (HRP)-conjugated protein was visualized using ECL Western Blotting Substrate (Millipore, Billerica, MA, USA). Densitometric analyses were performed using Image-Pro Plus (IPP) software (Media Cybernetics, USA).

### 2.10. Statistical Analysis

The experimental results were continuous variables, which were presented as mean ± SD. The entire database was statistically analyzed with SPSS22.0 software. One-way analysis of variance (ANOVA) was used to compare the differences between groups, and *p* < 0.05 was considered statistically significant. When significant between-group effects were present, post hoc comparisons were made using Fisher’s Least Significant Difference (LSD) (assumed equal variances) or Tamhane’s T2 method (assumed unequal variances).

## 3. Results

### 3.1. Macroscopic Evaluation of Ethanol-Induced Gastric Mucosal Lesions

**Figure 1:**
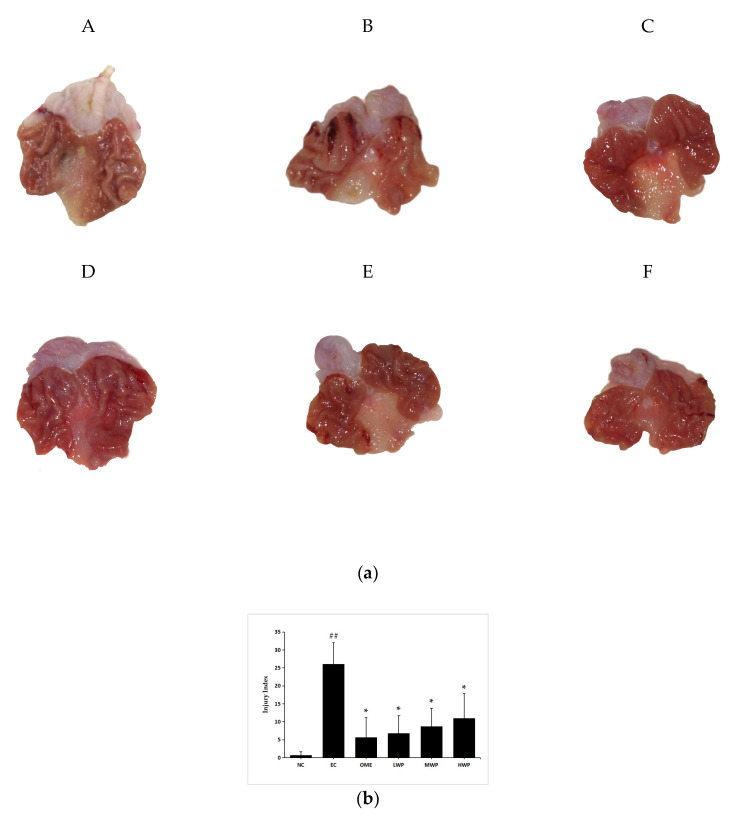
Macroscopical evaluation of protective effects of wheat peptides against gastric mucosal damage induced by ethanol. (**a**) Representative macroscopic photograph of the gastric mucosa in ethanol-induced gastric mucosal lesions in rats; (**b**) The gastric injury index of each group. Data are presented as mean ± SD (*n* = 10). Data are presented as mean ± SD (*n* = 10). ## *p* < 0.01 vs. normal control (NC) group, * *p* < 0.05 vs. ethanol model control (EC) group. A: NC, normal control; B: EC, ethanol control; C: omeprazole (OME), 20 mg/kgbw of omeprazole; D: low dose of wheat peptide (LWP), 0.1 g/kgbw of wheat peptide (WP); E: medium dose of wheat peptide (MWP), 0.2 g/kgbw of WP; F: high dose of wheat peptide (HWP), 0.4 g/kgbw of WP.

The macroscopic results displayed that absolute ethanol administration triggered several gastric mucosal lesions, appearing as diffuse edema, thick linear hemorrhagic zones, and multifocal erosions in the gastric mucosa layer along the long axis of the stomach (Figure 1a). However, the wheat peptides (WP) pretreated group and omeprazole groups extensively reduced the length and number of hemorrhagic stripes. Gastric damage was rare in the NC group. Meanwhile, the gastric injury index of the EC group was significantly higher than that of the NC group (*p* < 0.01, Figure 1b). Whereas, in comparison to the EC group, pretreatment with wheat peptides and omeprazole significantly reduced the injury index (*p* < 0.05).

### 3.2. Microscopic Morphology of the Gastric Mucosa

**Figure 2:**
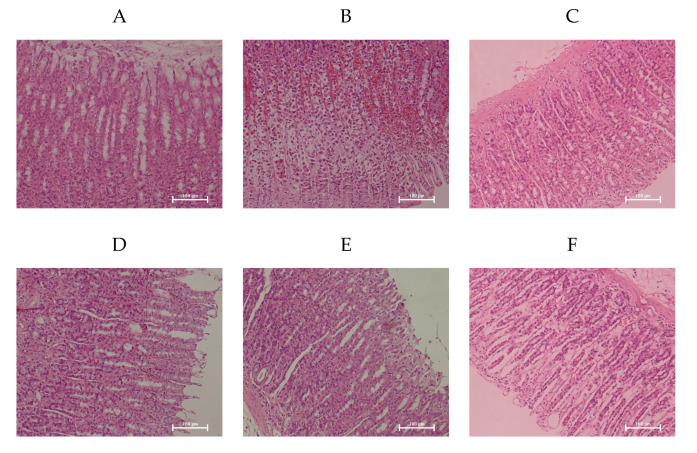
Wheat peptides alleviated ethanol-induced gastric histopathologic injury in rats. Photomicrographs of hematoxylin and eosin (magnification 200X) stained sections from gastric wall. EC group. (**A**): NC, normal control; (**B**): EC, ethanol control; (**C**): OME, 20 mg/kgbw of omeprazole; (**D**): LWP, 0.1 g/kgbw of WP; (**E**): MWP, 0.2 g/kgbw of WP; (**F**): HWP, 0.4 g/kgbw of WP.

Microscopic observation confirmed the ability of WP to attenuate ethanol-induced gastric mucosa damage contrasted with the NC group that showed the smooth and complete histological structure of gastric mucosa (Figure 2). Ethanol induced extensive damage of gastric mucosa that was characterized by sub-mucosa edema, hemorrhagic injury, mucosa degradation and distortion, destruction of the surface epithelium, and inflammatory cell infiltration. However, rats pretreated with WP showed the reduction in hemorrhagic lesions and leukocytic infiltrations of the gastric mucosa, which was comparable to the effects of omeprazole and consistent with the results of macroscopic observation.

### 3.3. Effects of Wheat Peptides on Gastric Mucosal Blood Flow (GMBF) 

#### 3.3.1. Changes of GMBF

**Figure 3:**
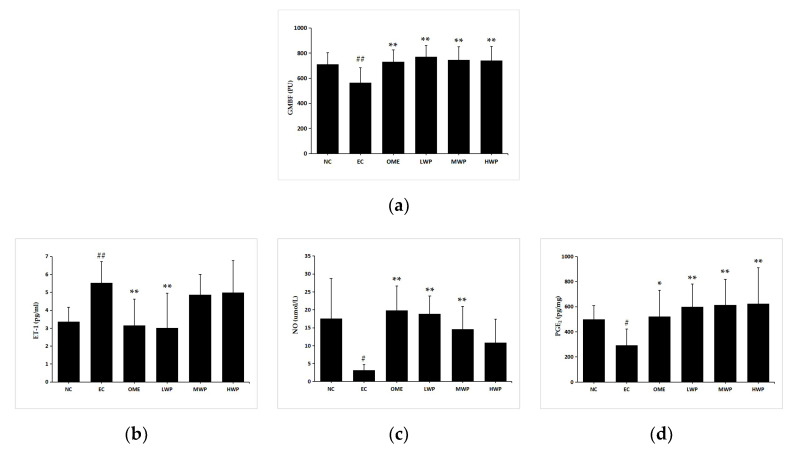
Effects of wheat peptides in improving gastric mucosal blood flow (GMBF) and relevant protection factors in rats with ethanol-induced gastric lesions. (**a**) The changes of GMBF measured by the laser doppler flowmeter instrument; (**b**) Plasma levels of endothelin (ET)-1; (**c**) Plasma levels of nitric oxide (NO); (**d**) Gastric prostaglandin E2 (PGE2). Data are presented as mean ± SD (*n* = 10). ## *p* < 0.01 vs. NC group, # *p* < 0.05 vs. NC group, ** *p* < 0.01 vs. EC group, * *p* < 0.05 vs. EC group. NC, normal control; EC, ethanol control; OME, 20 mg/kgbw of omeprazole; LWP, 0.1 g/kgbw of WP; MWP, 0.2 g/kgbw of WP; HWP, 0.4 g/kgbw of WP.

As shown in Figure 3a, induction of lesions by ethanol significantly decreased the GMBF measured by Laser Doppler Flowmeter (*p* < 0.01), in comparison to the NC group. Meanwhile, the administration of wheat peptides and omeprazole groups significantly increased the GMBF compared with the EC group (*p* < 0.01).

#### 3.3.2. Levels of NO, ET-1, and PGE2

Levels of the vasoconstrictor factor ET-1 were elevated remarkably in the EC group compared with the NC group (*p* < 0.01, Figure 3b). The administration of wheat peptides (0.1 g/kgbw) and omeprazole to the animals prominently suppressed excessive ET-1 production referenced to the ethanol-treated rats (*p* < 0.01), yet this only reached statistical significance in the LWP group. The imbalance between ET-1 and NO was proven to be relevant for vascular diseases. Levels of plasmal NO and gastric PGE2 in EC groups were significantly declined compared to the NC group (*p* < 0.05, Figure 3c,d). In opposition, pretreatment of rats with WP or omeprazole showed a significant increase in NO and PGE2 contents (*p* < 0.01 versus EC group), except for the NO level in the HWP group (*p* > 0.05). 

### 3.4. Effects of Wheat Peptides on Inflammatory Cytokines in Gastric Mucosa 

**Figure 4:**
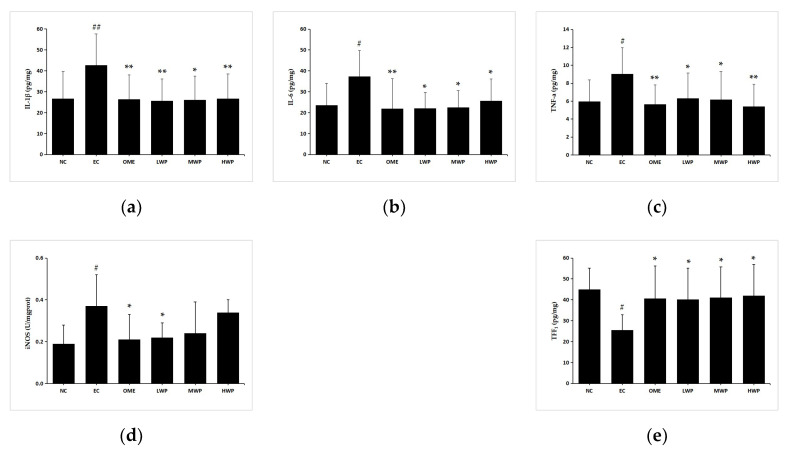
Effects of wheat peptides on tumor necrosis factor α (TNF-α), interleukin (IL)-1β, IL-6, inducible nitric oxide synthase (iNOS), and trefoil factor 1 (TFF1) in ethanol-induced gastric mucosal injury of rats. (**a**) Gastric IL-1β; (**b**) Gastric IL-6; (**c**) Gastric TNF-α; (**d**) Gastric iNOS; (**e**) Gastric TFF1. Data are presented as mean ± SD (*n* = 10). Data are presented as mean ± SD (*n* = 10). ## *p* < 0.01 vs. NC group, # *p* < 0.05 vs. NC group, ** *p* < 0.01 vs. EC group, * *p* < 0.05 vs. EC group. NC, normal control; EC, ethanol control; OME, 20 mg/kgbw of omeprazole; LWP, 0.1 g/kgbw of WP; MWP, 0.2 g/kgbw of WP; HWP, 0.4 g/kgbw of WP.

Pro-inflammatory cytokines play a crucial role in diverse inflammation-related diseases [22]. To elucidate the gastroprotective effects of wheat peptides on the ethanol-induced gastric mucosa injury rats, levels of pro-inflammatory cytokines were detected by enzyme-linked immunosorbent assay kits (Figure 4). After administration with ethanol, the secretion of gastric TNF-α, IL-1β, IL-6, and iNOS was significantly elevated (*p* < 0.05) in the EC groups as compared to the NC group. Nevertheless, pretreatment with wheat peptides and omeprazole groups significantly reversed the unfavorable effects of cytokines (*p* < 0.05 versus EC group). Levels of iNOS had no significant difference in the MWP and HWP groups (*p* > 0.05 versus EC group). In addition, a higher gastric TFF1 level, a protective factor for suppressing the inflammation of gastric mucosa, was observed when rats were supplemented with wheat peptides or omeprazole (*p* < 0.05 versus EC group). Thus, the aforementioned results suggested that WP could attenuate the gastric inflammation caused by ethanol in rats. 

### 3.5. Effects of Wheat Peptides on NF-κB Pathway in Gastric Mucosa

**Figure 5:**
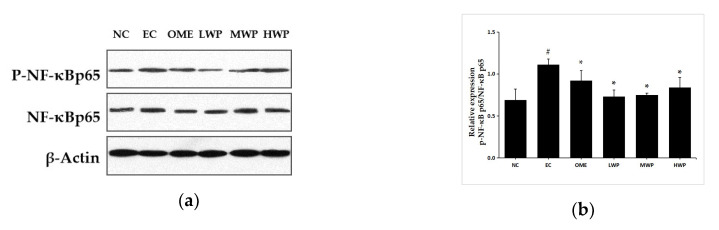
Effects of wheat peptides on the nuclear factor kappa-B (NF-κB) signal pathway in the ethanol-induced gastric lesions of rats. (**a**) Proteins levels of phosphorylated nuclear factor kappa-B (p-NF-κB) p65 and nuclear factor kappa-B (NF-κB) p65; (**b**) The relevant density of p-NF-κB p65/ NF-κB p65. Data are presented as mean ± SD (*n* = 3). ## *p* < 0.01 vs. NC group, # *p* < 0.05 vs. NC group, ** *p* < 0.01 vs. EC group, * *p* < 0.05 vs. EC group. NC, normal control; EC, ethanol control; OME, 20 mg/kgbw of omeprazole; LWP, 0.1 g/kgbw of WP; MWP, 0.2 g/kgbw of WP; HWP, 0.4 g/kgbw of WP.

To confirm whether the anti-inflammatory mechanisms of wheat peptides on ethanol-induced gastric lesions was related to the NF-κB signal pathway, the expression of p-NF-κB p65 and NF-κB p65 protein was assessed (Figure 5). As compared to the NC group, the protein expression of p-NF-κB p65 was significantly increased in the EC group. However, oral treatment with wheat peptides and omeprazole exhibited a significant reduction in the p-NF-κB p65 levels (*p* < 0.05).

## 4. Discussion

The major finding in this study is that wheat peptides could protect gastric mucosa from ethanol injury. Gastritis and gastric ulcers could be activated by various factors, such as inflammatory mediators, non-steroidal anti-inflammatory drugs (NSAIDs) and ethanol. Ethanol-induced gastric injury is a crucial experimental model for the evaluation of ethanol-induced pathological changes in gastric mucosa [23]. Ethanol and metabolite acetaldehyde damage the epithelial and vascular endothelial cells of gastric mucosa, inducing microcirculatory disturbance and hypoxia, along with propagation of the inflammatory cascade [24]. Wheat peptides have well established the properties of anti-oxidation and anti-inflammation in the ethanol-induced gastric injury model of rats. However, in our present study, pretreatment with wheat peptides significantly attenuated ethanol-induced gastric mucosal lesions in rats through improving gastric mucosal microcirculation, thereby inhibiting the inflammatory cascade mediated by the nuclear factor kappa-B (NF-κB) signaling pathway. The protection effects of wheat peptides for the gastric mucosa appear to be comparable to the classical antiulcer agent omeprazole. Consistent with the results of previous similar study on wheat peptides and fucoidan [21], an inverse dose-dependent alleviating effect of wheat peptides on gastric mucosal damage induced by ethanol was also found in the present research. Although wheat peptides are characterized with fast absorption and high efficiency, the process of digestion, absorption, and transportation of wheat peptides in rats is complicated [25,26,27]. We speculated that the ability of low-dose wheat peptides (0.1 g/kg) to bind to key transporters of target organs in rats is superior to higher doses; therefore, a better protective effect was found in the low-dose group rather than the two higher-dose groups in the present study. However, this still needs to be verified. In further study, we will explore the dose–effect relationship at the dosage lower than 0.1 g/kg, clarifying the optimal dose range of wheat peptides against mucosal injury of the digestive tract. 

Ethanol could dose-dependently damage the gastric mucosa, leading to desquamation of the mucosal epithelium and the release of inflammatory mediators. This results in the restriction of gastric blood flow, inflammatory cell infiltration, and the development of hemorrhagic gastritis. Histological results indicated that ethanol severely damaged the gastric mucosa, which is characterized by generalized edemas, hemorrhagic necrosis, loss of epithelial cells, and the infiltration of inflammatory cells in the mucosal and submucosal layers. This is consistent with previous research results [28]. We observed that wheat peptide or omeprazole obviously weakened these typical characteristics induced by ethanol, suggesting that the beneficial effects of wheat peptides on ethanol-induced gastric inflammation and lesions may be involved in inhibiting neutrophil migration into the gastric tissue.

Ethanol, a recognized necrotizing agent, induces inflammation response in the gastric mucosal epithelium cells and activates the nuclear factor kappa B (NF-κB) signaling pathway [29,30]. Pro-inflammatory cytokines and enzymes play an essential role in the ethanol-triggered gastric injury model of rats [31]. Tumor necrosis factor-α (TNF-α) is one of the most aggressive factors in the process of inflammation, injury, and carcinogenesis in gastric tissues [32]. High levels of mucosal TNF-α can aggravate gastric epithelial cell apoptosis triggered by ethanol [33]. TNF-α activates NF-κB and inducible nitric oxide synthase (iNOS) and stimulates the infiltration of neutrophils into the gastric mucosa [34]. Increased levels of IL-6 in gastric tissue stimulate phagocytes, lymphocytes, and neutrophils in inflammatory sites, activate oxidative stress, and produce toxic metabolites and lysosomal enzymes, leading to gastric mucosal lesions [35]. Interleukin (IL)-1β accelerates the accumulation of neutrophils and the release of inflammatory mediators, which is closely related to the severity of gastric ulcer triggered by ethanol [36]. The current data indicated that ethanol administration increased levels of iNOS, pro-inflammatory cytokines TNF-a, IL-1β, and IL-6 in the gastric tissue, which was in agreement with previous studies. The present study showed that pretreatment with wheat peptides remarkably declined the pro-inflammatory cytokines production in a rat model of ethanol-induced gastric lesions, speculating that the potential gastroprotective effects of wheat peptides may be related to its anti-inflammatory activity. 

Similarly, pro-inflammation cytokines TNF-a and IL-6 also activate NF-κB, which is the principal regulator of the transcription of several genes involved in inflammation during gastric injury formation [37]. The activation of NF-κB produced from macrophages after ethanol treatment regulates a large number of downstream inflammatory cytokines and mediator genes, thereby exaggerating the inflammation in stomach tissues [38]. Therefore, inhibiting the expression of NF-κB can make it a target for the treatment of inflammatory response conditions, thereby attenuating the gastric mucosal damage [39]. When ischemia occurs, activated NF-κB translocates to the nucleus and interacts with DNA response elements to initiate the transcription of target inflammatory genes. The p65 subunit of NF-κB is generally regarded as a marker of NF-κB activation and exerts a critical role in inflammation and immune regulation [40]. The results showed that ethanol administration up-regulated the transduction of phosphorylated nuclear factor kappa-B (p-NF-κB) p65 from the cytoplasm to the nucleus in the gastric tissue. The findings are in harmony with the previous literature [41]. In contrast, administrating wheat peptides decreased the protein expression of p-NF-kB p65 triggered by ethanol. The inhibition of p-NF-kB p65 for wheat peptides is an effective mechanism attenuating ethanol-induced gastric mucosal lesions, since the expression of multiple pro-inflammatory cytokines and enzymes is chiefly modulated by the NF-κB signaling pathway. 

The ingestion of ethanol causes microvascular endothelium impairment, reduces gastric mucosal blood flow (GMBF), and weakens the mucosal resistance, making it susceptible to damaging factors, even heightening the intensity of gastric damage [42]. Continuous blood flow lined with endothelial cells forms an endothelial “barrier” in gastric stomach [43]. In addition, nitric oxide (NO) and prostaglandin E2 (PGE2), which are endogenous molecules, are described as the second major mucosal defense mechanism, regulating the gastric mucosal acidity [44,45]. NO produced by endothelial cells has a direct vasodilator effect, inhibiting platelet aggregation and thrombosis, thus enhancing the blood flow of gastric mucosa [46,47]. Excessive NO produced by iNOS generates gastric mucosal damage and dysfunction [48]. PGE2 enhances mucosal blood flow and angiogenesis, and it accelerates epithelial wound repair and mucosal healing [49]. Endothelin (ET)-1 is a strong vasoconstrictor, and the increased release of ET-1 leads to dysregulation of the microcirculation [50]. It has been reported that ethanol stimulated the release of ET-1 in the gastric mucosa and caused a rapid, time-dependent increase in the level of ET-1 in plasma [51]. Previous study suggested that ethanol ingestion reduced the levels of PGE2 and NO cytoprotective moieties [52]. Wheat peptides have been reported to have the effects of promoting vasodilation, promoting the secretion of nitric oxide in vascular endothelial cells through an intermediate receptor. Moreover, TNF-α reduces gastric microcirculation around the damaged mucosa and delays healing because of the secondary release of the pro-inflammatory cytokines and the production of an acute phase of protein [53]. As a consequence, the anti-inflammatory effects of wheat peptides may correct gastric mucosal microcirculation disturbances. The current data demonstrated that wheat peptides administration caused a significant increase in GMBF, NO, and PGE2 levels, as well as a reduction in the level of ET-1. The study indicates that wheat peptides alleviate the impaired gastric microcirculation exposed to ethanol through improving the gastric mucosal blood flow and several endogenous defensive factors. 

It is well-identified that NO and PGE2 increase the secretion of mucus and bicarbonate in the maintenance of gastric mucosal integrity [54]. Trefoil factor 1 (TFF1) is a unique class of small molecule peptides that plays a vital role in gastric mucosal protection and post-injury repair, inhibiting the secretion of gastric acid and stimulating the proliferation of mucosal cells [55]. In the present study, ethanol exposure markedly reduced the levels of TFF1 in the gastric tissue. The expression of TFF1 mRNA has been reported to decrease in the experiment of acute gastric mucosal injury induced by absolute ethanol [56]. Interestingly, TFF1 could suppress the TNF-α-mediated activation of NF-κB [57]. However, preconditioning with wheat peptides exhibited a significant increase of TFF1, NO, and PGE2 levels induced by ethanol, suggesting that wheat peptides can be conducive to maintaining the integrity of gastric mucosa against the injurious effects of ethanol.

## 5. Conclusions

According to the results and literature, wheat peptides may be capable of protective effects against ethanol-induced gastric mucosal lesions in rats. These beneficial gastroprotective effects may be achieved by inhibiting the inflammation response mediated by the NF-kB signaling pathway, improving gastric mucosal blood flow, inducing endogenous molecules for cytoprotection, and maintaining the integrity of the gastric mucus barrier. Wheat peptides may become a potential bioactive substance for the adjuvant treatment of alcoholic gastritis. However, further research is necessary to elucidate the specific mechanisms of action of wheat peptides on ethanol-induced gastric mucosal lesions.

## Figures and Tables

**Table 1:** Scoring criteria for acute alcoholic injury.

Grade	1	2	3	4
Hemorrhagic spot	Each spot	-	-	-
Length of hemorrhagic stripe	1–5 mm	6–10 mm	10–15 mm	>15 mm
Width of hemorrhagic stripe	1–2 mm	>2 mm		
Injury Index = Hemorrhagic spot + Length Score + (Width score * 2)				
